# Perceptions of frontline staff regarding data collection methodologies used during the 2009 A H1N1 influenza immunization campaign in Canada

**DOI:** 10.1186/1471-2458-10-796

**Published:** 2010-12-30

**Authors:** Julie Foisy, Susan Quach, Christine L Heidebrecht, Jennifer A Pereira, Sherman D Quan, Maryse Guay, Julie A Bettinger, Shelley L Deeks, Stephanie Brien, Jeffrey C Kwong

**Affiliations:** 1 Department of Surveillance and Epidemiology, Ontario Agency for Health Protection and Promotion, Toronto, Canada; 2 University Health Network, Toronto, Canada; 3 Département des sciences de la santé communautaire, Université de Sherbrooke, Longueuil, Canada; 4 Institut national de santé publique du Québec, Montréal, Canada; 5 Agence de la santé et des services sociaux de la Montérégie, Longueuil, Canada; 6 Centre de recherche de l'Hôpital Charles LeMoyne, Longueuil, Canada; 7 University of British Columbia, Vaccine Evaluation Center, BC Children's Hospital, Vancouver, British Columbia, Canada; 8 Dalla Lana School of Public Health, University of Toronto, Toronto, Canada; 9 Department of Epidemiology, Biostatistics, and Occupational Health, McGill University, Montreal, Canada; 10 Institute for Clinical Evaluative Sciences, Toronto, Canada; 11 Department of Family and Community Medicine, University of Toronto, Toronto, Canada

## Abstract

**Background:**

During the 2009 H1N1 immunization campaign, electronic and hybrid (comprising both electronic and paper components) systems were employed to collect client-level vaccination data in clinics across Canada. Because different systems were used across the country, the 2009 immunization campaign offered an opportunity to study the usability of the various data collection methods.

**Methods:**

A convenience sample of clinic staff working in public health agencies and hospitals in 9 provinces/territories across Canada completed a questionnaire in which they indicated their level of agreement with seven statements regarding the usability of the data collection system employed at their vaccination clinic. Questions included overall ease of use, effectiveness of the method utilized, efficiency at completing tasks, comfort using the method, ability to recover from mistakes, ease of learning the method and overall satisfaction with the method. A 5-point Likert-type scale was used to measure responses.

**Results:**

Most respondents (96%) were employed in sites run by public health. Respondents included 186 nurses and 114 administrative staff, among whom 90% and 47%, respectively, used a paper-based method for data collection. Approximately half the respondents had a year or less of experience with immunization-related tasks during seasonal influenza campaigns. Over 90% of all frontline staff found their data collection method easy to use, perceived it to be effective in helping them complete their tasks, felt quick and comfortable using the method, and found the method easy to learn, regardless of whether a hybrid or electronic system was used.

**Conclusions:**

This study demonstrates that there may be a greater willingness of frontline immunization staff to adapt to new technologies than previously perceived by decision-makers. The public health community should recognize that usability may not be a barrier to implementing electronic methods for collecting individual-level immunization data.

## Background

Collecting individual-level information at the point of vaccination enables timely assessment of vaccination coverage, effectiveness and safety at the population level[1].

Vaccine providers must consider many factors for optimizing data collection, including available financial and human resources, and the impact that the approach will have on users at all levels of the system. As technology has evolved and the value of electronic patient records is increasingly recognized, electronic means of collecting and storing health data are becoming more viable. However, usability constraints have been identified as a potential obstacle to implementing novel technologies[2].

During the 2009 A H1N1 influenza immunization campaign, paper, electronic, and hybrid systems were employed in clinics across Canada. Because different systems were used across the country, the 2009 immunization campaign offered an opportunity to study the efficiency and usability of these data collection methods. The objective of this study was to determine the perceptions held by frontline clinic staff of H1N1 immunization data collection methods used in Canada.

## Methods

### Setting

The Public Health Agency of Canada/Canadian Institutes of Health Research Influenza Research Network (PCIRN) Vaccine Coverage Theme conducted an on-site assessment of pandemic immunization data collection among a sample of public health agencies and hospitals that provided influenza immunization across Canada from October to December 2009. All public health jurisdictions in Canada were contacted by email and invited to participate. The front-line staff perceptions survey was administered as part of a larger on-site assessment that included observing data collection methodologies, and measuring the time spent by front-line immunization staff to record pandemic immunization data. Results from the time and motion study, a part of the larger on-site assessment, have been published elsewhere[3]. This report summarizes the questionnaire that was administered to clinic staff to assess usability of the data collection methods. Details of the methods and results for the on-site assessment objectives have been described elsewhere[4]. Ethics approval was obtained from the University of Toronto's Health Sciences Research Ethics Board (REB) and other jurisdictions' REBs as required.

### Questionnaire Development

The IBM Computer System Usability Questionnaire (CSUQ) is a validated 19-item instrument that was originally developed to measure users' satisfaction with the usability of computer systems in non-laboratory settings[5]. We modified the questionnaire and retained 7 questions that were directly applicable to both paper and electronic immunization collection systems. Questions included overall ease of use, effectiveness of the method utilized, efficiency at completing tasks, comfort using the method, ability to recover from mistakes, ease of learning the method and overall satisfaction with the method. Using a Likert-type scale, response choices included strongly disagree, disagree, neither agree nor disagree, agree, strongly agree and not applicable. Additional questions addressed respondent's position, responsibilities, number of years with said responsibilities, and location of clinic. A copy of the questionnaire can be found in the additional files.

### Administration of the Questionnaire

The questionnaire was completed by a convenience sample of frontline staff at immunization clinics that were visited between October 27 and December 17, 2009. Participants were approached by a member of the research team and asked to provide informed consent prior to being observed, as part of the on-site assessment. Participants were also asked to complete the questionnaire at any point during the day, with assurance of the anonymity of their responses. The questionnaire was then given to the participants and collected from them by the end of the day.

### Statistical Analysis

Since participants generally performed a limited range of tasks in the immunization process (e.g., administrative staff may have only registered clients using an electronic method, while nurses performed immunization and related documentation using a paper method), questionnaire responses were dichotomised according to the data collection *method *used for the task(s) (i.e., electronic vs. paper). Analyses consisted of frequency distributions. Statistical significance testing was conducted using Fisher's exact test to identify differences between those using electronic and paper methods (all staff combined, nurses and administrative clerks separately). Exact 95% confidence limits were calculated. Analyses were performed using STATA version 10.0[6].

## Results

Of the 165 organizations contacted across Canada, 38 (23%) (with 79 physically distinct immunization clinic sites across Canada) agreed to participate and 300 front-line staff responded to the survey. The number of staff who refused to respond to the survey was not tracked; however it is believed to be very few.

The characteristics of the participating frontline staff can be found in Table 1. The majority of respondents were employed in sites operated by public health (96%). Respondents included 186 nurses and 114 administrative staff, among whom 90% and 47% respectively used a paper method to perform data collection tasks. Over 52% of respondents had a year or less of experience with immunization-related tasks during seasonal influenza campaigns; the number of years of experience ranged from 0 to 32.

**Table 1:** Characteristics of respondents

Characteristics		n (%)
**Total Respondents**		**300 (100)**

**Type of Site**		

	Public Health	287 (95.7)

	Hospital	13 (4.3)

**Method Used**		

	Electronic	80 (26.7)

	Paper	220 (73.3)

**Nurses**		**186 (100)**

	Using electronic methods	19 (10.2)

	Using paper methods	167 (89.8)

	Median years of experience with immunization task (range)	6 (0 - 32)

**Administrative clerks**		**114 (100)**

	Using electronic methods	61 (53.5)

	Using paper methods	53 (46.5)

	Median years of experience (range)	1 (0 - 29)

Frontline staff found the data collection system used in their clinic, whether electronic or paper, to be highly acceptable (Table 2). Among all frontline staff using an electronic method, 96% felt comfortable with the approach being used. Ninety-six percent of frontline staff using an electronic method and 94% of staff using a paper method found the method easy to learn. Ninety-eight percent of electronic method users and 91% of paper method users strongly agreed/agreed with the statement: "Overall I am satisfied with this method". No statistically significant differences were found between users of electronic and paper methods (nurses, administrative clerks or all staff combined), however, we observed a trend favouring electronic over paper methods in the overall satisfaction question for all staff combined (p = 0.08).

**Table 2:** Perceptions of frontline staff by position and method

	Nurses		Administrative clerks		All staff combined	
	**Strongly agree/agree % (95% CI)**		**Strongly agree/agree % (95% CI)**		**Strongly agree/agree % (95% CI)**	

**Statement**	**Electronic Method** **(n = 19)**	**Paper Method** **(n = 167)**	**Electronic Method** **(n = 61)**	**Paper Method** **(n = 53)**	**Electronic Method** **(n = 80)**	**Paper Method** **(n = 220)**

It was easy to use this data collection method.	94.7% (74.0-99.9)	91.0% (85.6-94.9)	96.7% (88.7-99.6)	84.9% (72.4-93.3)	96.3% (89.4-99.2)	89.5% (84.7-93.3)

I could effectively complete my tasks using this method.	100% (82.4-100)	94.0% (89.3-97.1)	96.7% (88.7-99.6)	92.5% (81.8-97.9)	97.5% (91.3-99.7)	93.6% (89.6-96.5)

I was able to complete my tasks quickly using this method.	94.7% (74.0-99.9)	89.2% (83.5-93.5)	93.4% (84.1-98.2)	84.9% (72.4-93.3)	93.8% 86.0-97.9)	88.2% (83.2-92.1)

I felt comfortable using this method.	94.7% (74.0-99.9)	95.2% (90.8-97.9)	96.7% (88.7-99.6)	90.6% (79.3-96.9)	96.3% (89.4-99.2)	94.1% (90.1-96.8)

It was easy to learn to use this method.	89.5% (66.9-98.7)	93.4% (88.5-96.7)	98.4% (91.2-100)	96.2% (87.0-99.5)	96.3% (89.4-99.2)	94.1% (90.1-96.8)

Whenever I make a mistake using this method, I can recover easily and quickly.	84.2% (60.4-96.6)	87.4% (81.4-92.0)	93.4% (84.1-98.2)	90.6% (79.3-96.9)	91.3%(82.8-96.4)	88.2% (83.2-92.1)

Overall, I am satisfied with this method.	100% (82.4-100)	91.6% (86.3-95.3)	96.7% (88.7-99.6)	86.8% (74.7-94.5)	97.5% (91.3-99.7)	90.5% (85.8-94.0)

## Discussion

Our study indicates that frontline workers are highly satisfied with the data collection methods used at vaccination clinics regardless of whether it was paper-based or electronic. Over 90% of all frontline staff found their data collection method quick and easy to use, perceived it to be effective in helping them complete their tasks, felt comfortable using the method, and found it easy to learn.

In a national study conducted prior to the H1N1 campaign[7] decision makers identified training of frontline staff as a perceived barrier to implementing electronic methods of collecting immunization data. Familiarity with a system and increased training raise users' acceptability of a novel system[8]. One of the electronic systems used in Canada - and the one used by the majority of observed organizations - was developed and rolled out in a very short period of time. Although users received training for this new system, it may not have been as extensive as it would have been during a regular influenza season due to time constraints associated with the urgent vaccine delivery schedule. Our results show that although most users had minimal experience with this electronic system, they nonetheless found the computer-based tasks highly acceptable. In a separate survey, 69% of nurses indicated that they had received adequate training prior to use of this novel system[9].

This study had several limitations. First, although the IBM survey has been validated for new computer users it may not be directly applicable to users of paper-based systems. It has also not been validated specifically to assess usability of immunization data collection methods by healthcare staff. Many may not have used both paper and electronic methods and therefore would not have been in a position to directly compare them. Finally, the questionnaire was only completed by frontline staff who were observed for the time and motion study, which mainly consisted of those using a paper method. Because of this limited sample, our comparisons may have inadequate power to detect a true difference between methods.

## Conclusion

This study suggests that there may be a greater willingness of frontline immunization staff to adapt to new technologies than previously perceived by decision-makers. The high acceptability for both electronic and paper-based methods illustrates that frontline staff are content with either method, regardless of the novelty. The public health community should recognize that usability may not be a barrier to implementing electronic methods for collecting individual-level immunization data. Utilizing electronic methods for collecting individual-level data offers the possibility for data to be analyzed and applied quickly for decision-making purposes, which could result in timely assessment of vaccine coverage, effectiveness and safety.

## Competing interests

The authors declare that they have no competing interests.

## Authors' contributions

All authors were involved with the study design. JF, SQ, CLH and JAP collected the data. JF and SQ analyzed the data. JF drafted the manuscript with contributions from CLH, JAP, SQ, SDQ, SLD, MG, JAB, SB, and JCK. All authors read and approved the manuscript.

## Pre-publication history

The pre-publication history for this paper can be accessed here:

http://www.biomedcentral.com/1471-2458/10/796/prepub

## Supplementary Material

Additional file 1 **User Perceptions Questionnaire**. A copy of the "User Perceptions Questionnaire" that was used for this study. Click here for file
