# Supplementary material for: Perceptions of frontline staff regarding data collection methodologies used during the 2009 A H1N1 influenza immunization campaign in Canada
Source: BMC Public Health. 2010 Dec 30;10:796. doi: 10.1186/1471-2458-10-796 (PMC3024979; doi:10.1186/1471-2458-10-796)
Supplement: Additional file 1 — User Perceptions Questionnaire. A copy of the "User Perceptions Questionnaire" that was used for this study. [file 1471-2458-10-796-S1.PDF]

Ontario

Agency for Health
Protection and Promotion

Perceptions of Pandemic (H1N1) 2009 Influenza
Patient Data Collection Methods Questionnaire

The Public Health Agency of Canada/Canadian Institutes of Health Research Influenza Research Network (PCIRN) is conducting a questionnaire to evaluate the different systems being used across Canada to collect pandemic (H1N1) 2009 influenza patient immunization data. To collect this patient data, you may be using an electronic system, a purely paper-based system, or a paper system with a later transfer into an electronic system.

We would appreciate if you would complete the below survey, which will take you approximately 5 – 10 minutes. By completing this questionnaire, you will be providing your opinions regarding the system you are using. Your responses will help us understand what aspects of the method you are satisfied with and the aspects that could be improved upon to facilitate data collection.

Please indicate your position, by checkmark below:

- ☐ Administrative staff
- ☐ Physician
- ☐ Vaccination nurse
- ☐ Post-vaccination nurse
- ☐ Other; please specify:___________________________

Please indicate your responsibilities, by checkmark below (more than one may apply):

- ☐ Patient registration
- ☐ Medical history collection
- ☐ Medical history review
- ☐ Vaccine administration and record-keeping
- ☐ Preparation of Proof of Vaccine Administration

Please indicate the number of years you have had the above responsibility(ies) during seasonal influenza seasons:_______

Please indicate your site type, by checkmark below:

- ☐ Public health site
- ☐ Hospital or other healthcare institution
- ☐ Physician office
- ☐ Other

City:______________________________ Province: _______________________

Please indicate the type of Data Collection Method used at your site, by checkmark below:

- ☐ Electronic system
- ☐ Paper-based system
- ☐ Hybrid (paper-based system, transferred into electronic form)

Number of years you have used this data collection method at your site: ________

Ontario
Agency for Health
Protection and Promotion

Perceptions of Pandemic (H1N1) 2009 influenza
Patient Data Collection Methods Questionnaire (page 2)

As you read each statement, please consider all of the tasks that you complete at your immunization site, using your data collection method. Then, **indicate your level of agreement with the statement by circling the appropriate response**. If a statement does not apply to you, please circle N/A.

1- It was easy to use this data collection method.

Strongly Disagree	Disagree	Neither Agree nor Disagree	Agree	Strongly Agree	N/A

2- I could effectively complete my tasks using this method.

Strongly Disagree	Disagree	Neither Agree nor Disagree	Agree	Strongly Agree	N/A

3- I was able to complete my tasks quickly using this method.

Strongly Disagree	Disagree	Neither Agree nor Disagree	Agree	Strongly Agree	N/A

4- I felt comfortable using this method.

Strongly Disagree	Disagree	Neither Agree nor Disagree	Agree	Strongly Agree	N/A

5- It was easy to learn to use this method.

Strongly Disagree	Disagree	Neither Agree nor Disagree	Agree	Strongly Agree	N/A

6- Whenever I make a mistake using this method, I can recover easily and quickly.

Strongly Disagree	Disagree	Neither Agree nor Disagree	Agree	Strongly Agree	N/A

7- Overall, I am satisfied with this method.

Strongly Disagree	Disagree	Neither Agree nor Disagree	Agree	Strongly Agree	N/A

Please return your completed questionnaire to the Research Associate.
Thank you!
